# Evaluation of Two Outlier-Detection-Based Methods for Detecting Tissue-Selective Genes from Microarray Data

**Published:** 2007-05-01

**Authors:** Koji Kadota, Tomokazu Konishi, Kentaro Shimizu

**Affiliations:** 1 Graduate School of Agricultural and Life Sciences, The University of Tokyo, 1-1-1 Yayoi, Bunkyo-ku, Tokyo 113-8657, Japan; 2 Faculty of Bioresource Sciences, Akita Prefectural University, Shimoshinjyo, Nakano, Akita 010-0195, Japan

**Keywords:** microarray, tissue selectivity, differential expression, AIC

## Abstract

Large-scale expression profiling using DNA microarrays enables identification of tissue-selective genes for which expression is considerably higher and/or lower in some tissues than in others. Among numerous possible methods, only two outlier-detection-based methods (an AIC-based method and Sprent’s non-parametric method) can treat equally various types of selective patterns, but they produce substantially different results. We investigated the performance of these two methods for different parameter settings and for a reduced number of samples. We focused on their ability to detect selective expression patterns robustly. We applied them to public microarray data collected from 36 normal human tissue samples and analyzed the effects of both changing the parameter settings and reducing the number of samples. The AIC-based method was more robust in both cases. The findings confirm that the use of the AIC-based method in the recently proposed ROKU method for detecting tissue-selective expression patterns is correct and that Sprent’s method is not suitable for ROKU.

## Introduction

The majority of microarray studies have focused on the detection of differentially expressed genes. Of these, tissue-selective genes for which expression in a single or small number of tissues is significantly different than in other tissues have attracted great interest due to their value in revealing the biological and physiological functions of tissues and organs at the molecular level (Kadota et al. 2006; Liang et al. 2006).

Numerous methods have been used to detect tissue-selective genes in microarrays (Greller and Tobin, 1999; Pavlidis and Noble, 2001; Kadota et al. 2003a; Schug et al. 2005; Ge et al. 2005; Yanai et al. 2005; Liang et al. 2006; Kadota et al. 2006). Of these, A recent study (ROKU; Kadota et al. 2006) demonstrated the effectiveness of using both Shannon entropy for ranking genes on the basis of their tissue selectivity (Schug et al. 2005) and an outlier-detection-based method for identifying tissues in which a gene is selective (the AIC-based method; Kadota et al. 2003a). However, it did not clarify why an AIC-based method was used even though other types of outlier-detection-based methods are applicable (Kadota et al. 2006). For example, Sprent’s non-parametric method could be used (Ge et al. 2005).

We have now evaluated and compared two outlier-based methods previously used for the detection of tissue-selective genes: the AIC-based method (Kadota et al. 2003a) and Sprent’s non-parametric method (Ge et al. 2005). Their outputs greatly vary mainly with changes in two factors. One is the maximum number of outlier candidates. For example, the AIC-based method sets this parameter to half the sample number interrogated; it can of course be set to other numbers. The other is the number of samples in the dataset. Researchers may subtract (or add) samples from a dataset if the data quality is under a post-determined threshold (or because other samples are added). The outputs for the common parts from two slightly different datasets can differ. Of course, we want to use a method for which the output is robust against changes in both factors. The two outlier-detection-based methods were evaluated in terms of these two factors.

## Methods

### Gene expression data

Expression data for normal human tissues were obtained from a dataset consisting of data for 36 various types of tissues in Affymetrix high-density oligonucleotide microarrays representing 22283 clones and controls (http://www.genome.rcast.u-tokyo.ac.jp/normal/). The raw (probe-level) data were processed using the SuperNORM algorithm (Konishi, 2004 and 2006) and log_2_ transformed.

### Detecting specific tissues using the AIC-based method

Detection of specific tissues using the AIC-based method (Kadota et al. 2003a) is performed as follows: (i) normalize gene vector *x* = (*x*_1_, *x*_2_, …, *x_N_*) for *N* tissues (*x*_1_ < *x*_2_ … < *x_N_*) by subtracting the mean and dividing by the standard deviation (SD); (ii) calculate statistics $U=n\times \text{log}\sigma +\sqrt{2}\times s\times (\text{log}n!)/n$ for various combinations of outlier candidates, where *n* and *s* denote the numbers of non-outlier and outlier candidates and σ denotes the SD of the observations of the *n* non-outlier candidates; and (iii) regard tissues corresponding to outliers detected in the combination of minimum *U* as specific. The maximum number, *N_max_*, of outlier candidates was originally set to *N*/2 (Kadota et al. 2003a). We analyzed the effect of changing *N_max_*. The R code is available in the additional file 2.

### Detecting specific tissues using Sprent’s method

Detection of specific tissues using Sprent’s non-parametric method (Ge et al. 2005) is performed as follows: (i) normalize gene vector *x* = (*x*_1_, *x*_2_, …, *x_N_*) for *N* tissues by subtracting the median and dividing by the median absolute deviation (MAD); (ii) regard tissues corresponding to absolute values >*k* as specific. Parameter *k* was originally set to 5 (Ge et al. 2005). We analyzed the effect of changing *k*.

## Results and Discussion

The purpose of this study was to compare two outlier-detection-based methods (the AIC-based method and Sprent’s non-parametric method) for the detection of tissues in which a gene is selective. Compared to other statistical methods excluding ROKU, which uses the AIC-based method (Kadota et al. 2006), both methods have two advantages. First, they can treat equally various types of tissue-selective genes: (a) ‘up-type’ genes selectively over-expressed in a single or small number of tissues, (b) ‘down-type’ genes selectively under-expressed in some tissues, and (c) ‘mixed-type’ genes selectively over- and under-expressed in some tissues (Kadota et al. 2006). Second, they can extract genes whose expression is considerably different only in arbitrarily selected tissues. Other methods such as template matching (Pavlidis and Noble, 2001) and Schug’s *Q*-statistic (Schug et al. 2005) sometimes detect genes considerably different in other tissues in addition to the objective tissue (Kadota et al. 2003a; Kadota et al. 2006).

Although neither method can rank genes on the basis of their overall tissue selectivity, ROKU can compensate for this by adding an entropy-based score for individual genes (Kadota et al. 2006). For ROKU users who want to detect various types of tissue-selective patterns, the remaining issue is whether another published method (Sprent’s method; Ge et al. 2005) is suitable for ROKU. Fortunately, the two methods have two common characteristics: (i) the same output format and (ii) only one parameter can affect the output (*N_max_* for the AIC-based method and *k* for Sprent’s method). These similarities facilitate direct comparison with no modifications.

Here we examine the effects of (1) different parameter settings and (2) a reduced number of samples on robustness. We do this using the expression data for 22283 clones and 36 samples. We first present an example using a hypothetical expression vector for ten tissues, *x* = (12, 51, 52, 54, 57, 59, 60, 63, 85, 88) and then evaluate the two methods using actual microarray data. Both methods output a vector (consisting of 1 for over-expressed outliers, −1 for under-expressed outliers, and 0 for non-outliers) that corresponds to the input expression vector. We only need compare these outlier vectors.

### Effect of different parameter settings

**Figure 1:**
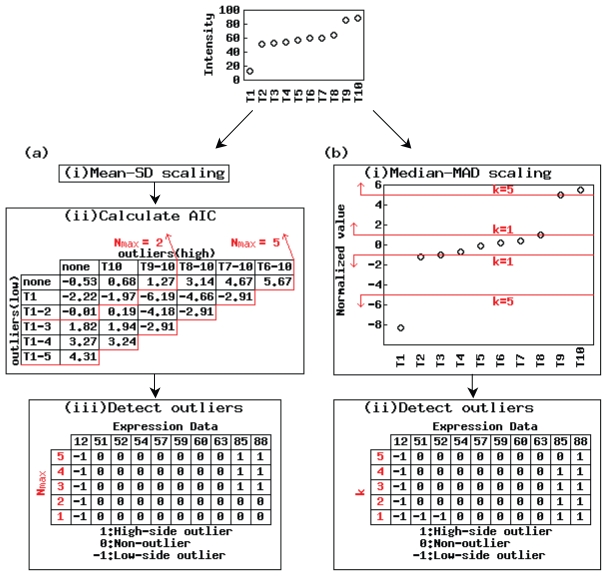
Calculation of outlier vectors using different parameter settings for hypothetical input vector The procedure for (a) the AIC-based method and (b) Sprent’s method are shown. Changing the parameter settings changed the outlier vectors.

The outlier vectors produced using outlier-detection-based methods vary with the parameters (*N_max_* for the AIC-based method and *k* for Sprent’s method) (Figure 1). In general, the number of detected outliers (the number of nonzero elements in the outlier vector) tends to be lower when *N_max_* is small and *k* is large. For example, reducing *N_max_*, which is the maximum number of outlier candidates, from 5 to 1 produced two different outlier vectors: (−1, 0, 0, 0, 0, 0, 0, 0, 1, 1) for *N_max_* = 3 to 5 and (−1, 0, 0, 0, 0, 0, 0, 0, 0, 0) for *N_max_* = 1 and 2 (Figure 1a). This is not surprising since the latter values of *N_max_* are less than the number of outliers detected using the former values of *N_max_* (1 or 2 <3). There is also some variation in the outlier vectors produced using different values of parameter *k* in Sprent’s method (Figure 1b).

For the hypothetical vector, the two outlier-detection-based methods with the default parameter settings (*N_max_* = *k* = 5) produce different outlier vectors. The difference is whether the second highest observation (the value of “85”) is detected as an over-expressed outlier (the AIC-based method) or a non-outlier (Sprent’s method). Since we designed the original hypothetical expression vector to have three significantly different observations than in the others (the same as the outlier vector obtained using the AIC-based method), the observation should be detected as an over-expressed outlier. Some researchers, however, disagree with our judgment and think, for example, there is only one tissue (T1) in which the hypothetical vector is selective. The final decision about tissue selectivity thus suffers from some subjectivity. Accordingly, we would be unable to determine which of the alternative methods performs better even if demonstrations for many hypothetical expression vectors and many actual vectors were provided. Figure 1 merely presents an example of producing different outlier vectors with different parameter settings.

**Figure 2:**
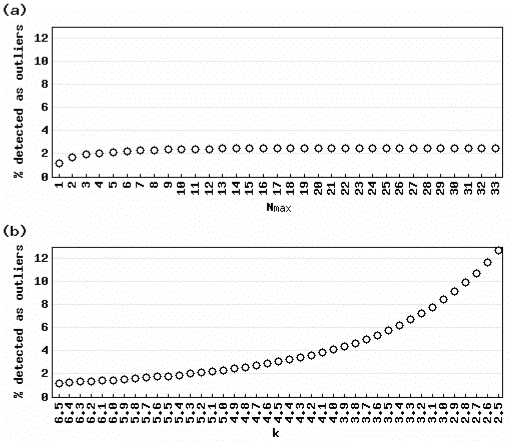
Effect of different parameter settings for actual data Percentages of detected outliers using (a) the AIC-based method and (b) Sprent’s method are shown. Note that those for the AIC-based method were more invariant than for Sprent’s method.

Figure 2 shows the average percentage of detected outliers for various values of *N_max_* (Figure 2a) and *k* (Figure 2b) when actual gene expression vectors for 36 normal human tissues (Ge et al. 2005) were analyzed. The results with the default parameter settings (*N_max_* = *N*/2 = 18; *k* = 5) yielded similar average percentages: 2.43% for the AIC-based method and 2.32% for Sprent’s method. Clearly, the percentages for the AIC-based method were insensitive to changes in the parameter value while those for Sprent’s method were sensitive. For example, changing *N_max_* from 9 (N*1/4) to 27 (N*3/4) yielded a difference of 0.06% (2.43– 2.37%) (Figure 2a), while changing *k* from 4.0 to 6.0 yielded a difference of 2.64% (4.11–1.47%) (Figure 2b). Although the ranges for the AIC-based method (9–27) and Sprent’s method (4.0–6.0) are not directly comparable, these parameters are possible. These results suggest that researchers who want a method for detecting tissues in which a gene is selective that is insensitive to variations in these parameters should use the AIC-based method. The “outlier matrix” (consisting of 1 for over-expressed outliers, −1 for under-expressed outliers, and 0 for non-outliers) that corresponds to the actual gene expression matrix when the AIC-based method is used with the default parameter setting is available in the additional file 1.

An interesting exercise is to change the $\sqrt{2}$ in the AIC criterion for detecting outliers to other values such as 1 or 2 though the original equation ( $U=n\times \text{log}\sigma +\sqrt{2}\times s\times (\text{log}n!)/n$) has a solid theoretical basis (Ueda T, 1996; Kadota et al. 2003a; Kadota et al. 2003b). A decrease (or increase) in the weight for the penalty results in an increased (or decreased) number of outliers. Changing $\sqrt{2}$ to 1 (or 2) with the default value of *N_max_* (18) yielded 5.19% (or 1.13%) for the average percentage of detected outliers. The AIC-based method remained robust against changes in *N_max_* when these other weights were used (data not shown).

### Effect of reduced number of samples

In addition to the effect of different parameter settings, outlier vectors could also vary with the addition or reduction of samples even when the same parameter values are used. To examine the effect of reducing the number of samples, we generated *N* leave-one-out input vectors consisting of (*N*−1) samples from an expression vector originally consisting of *N* samples. Consider, for example, a hypothetical vector consisting of ten observations. Ten leave-one-out input vectors, each of which has nine observations, can be analyzed. If the method is good, the ten leave-one-out output vectors should be the same as the original output vector of ten observations.

**Figure 3:**
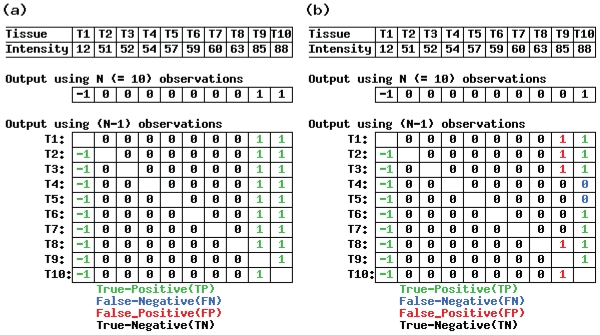
Example of leave-one-out outlier detection (LOOOD) for a hypothetical input vector The output vectors were obtained using (a) the AIC-based method and (b) Sprent’s method with default parameter values (*N_max_* = *k* = 5).

Figure 3 shows the results of the “leave-one-out outlier detection” (LOOOD) analysis for the hypothetical vector using (a) the AIC-based method and (b) Sprent’s method, with the default parameter settings (*N_max_* = *k* = 5). Clearly, the AIC-based method is more robust against a reduction in the number of samples, at least for this hypothetical expression vector.

To examine the two methods further using actual data, we defined a basis for evaluation as follows: (i) the outlier vector obtained from the original vector (not a leave-one-out vector) is “true,” (ii) the outliers (“−1” or “1”) in the outlier vector are “positive,” and (iii) the non-outliers (“0”) are “negative.” Accordingly, the LOOOD results give rise to four quantities:

True positive (TP): outliers that are detected as outliers in the outlier vector obtained from the original expression vector consisting of *N* observations

True negative (TN): non-outliers that are detected as non-outliers in the original outlier vector

False positive (FP): outliers that are detected as non-outliers in the original outlier vector

False negative (FN): non-outliers that are detected as outliers in the original outlier vector

For example, the values for Sprent’s method (Figure 3b) were TP = 16, TN = 67, FP = 5, and FN = 2. Zviling et al. (2005) stated that any single number that represents the power of the method must account for all the categories listed above. We define two such numbers: “accuracy” = (TP + TN)/(TP + TN + FP + FN) and “Matthews correlation coefficient (MCC)” = (TP*TN − FP*FN)/((TP + FN)*(TN + FP)*(TP + FP)*(TN + FN))^1/2^ (Matthews, 1975). Accuracy represents the fraction of the unchanged vectors among LOOOD test, and MCC represents the correlation between the original vector and the LOOOD results when the Pearson correlation coefficient is used. These statistics can take values in the following ranges: 0 ≤ accuracy ≤1; −1 ≤ MCC ≤1. The higher the value, the greater the robustness against a reduction in the number of samples. The LOOOD results for the hypothetical vector and Sprent’s method were accuracy = 92.22% and MCC = 77.50% (Figure 3b); for the AIC-based method (Figure 3a), they were accuracy = MCC = 100% since FP = FN = 0.

**Figure 4:**
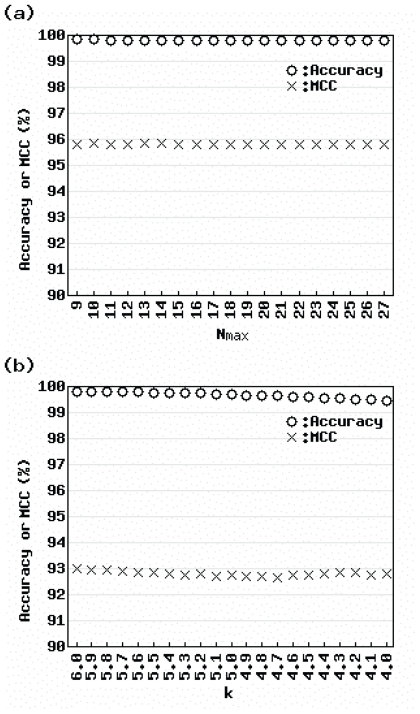
Effect of reduced number of samples for actual data Accuracy (circles) and Matthews correlation coefficient (crosses) are shown. Parameter values shown include the range of values likely used in practice.

Figure 4 shows the LOOOD results for actual data using (a) the AIC-based method and (b) Sprent’s method. Accuracy and MCC were calculated for each parameter value (*N_max_* = 9 – 27 and *k* = 4.0 – 6.0) around the default values (*N_max_* = 18 and *k* = 5). Obviously, the values for the AIC-based method were higher than those for Sprent’s method. We verified these results by varying the value of *N* in leave-*N*-out outlier detection (data not shown). These results suggest that the AIC-based method is less affected by slight changes in the input vector than Sprent’s method.

As mentioned above, objective comparison of methods for detecting tissue-selective patterns is understandably difficult. We know of only two reports in which the authors explicitly compared their method to other methods using the same dataset: (i) Kadota et al. (2003a) reported that the AIC-based method is superior to template matching and ANOVA, and (ii) Kadota et al. (2006) reported that ROKU can compensate for the disadvantages of the AIC-based method and of the entropy-based method proposed by Schug et al. (2005). The reports on the consistency between the results for a reduced number of samples and those for all the samples (Broberg P, 2003; Breitling et al. 2004) are of limited value because the results for all the samples were assumed to be correct (Jeffery et al. 2006). There is of course no guarantee, but it is probably safe to say that a higher number of samples should produce better results. Therefore, we still appreciate the advantages of the AIC-based method compared to Sprent’s method.

## Conclusion

We compared two outlier-detection-based methods previously used for the detection of tissue-selective genes. The AIC-based method was found to be better than Sprent’s non-parametric method in terms of robustness of the output against (1) a change in the parameter settings and (2) a reduction in the numbers of samples. These findings suggest that the use of the AIC-based method rather than Sprent’s method in the recently proposed ROKU method for detecting tissue-selective expression patterns was correct.

More work remains to be done. First, while the AIC-based method has clear advantages compared to Sprent’s method, the Bayesian information criterion (BIC) should also be applicable. It would be interesting to develop a BIC-based method and compare its performance to that of the AIC-based method. Second, the approach used here is not suitable for comparing ROKU with other methods such as the Tukey-Kramer’s honestly significant difference test due to their different output formats and the lack of genuine tissue-selective genes. We plan to develop a better approach for comparing a number of methods for detecting tissue-selective expression patterns.

## Additional File

Additional file 1 (additional.txt) – includes all data analyzed using AIC-based method for dataset of Ge et al. (2005). For the original gene expression matrix, an outlier matrix (consisting of 1 for over-expressed outliers, −1 for under-expressed outliers, and 0 for non-outliers) is provided. It also contains an entropy score (*H*′) measured by ROKU.

Additional file 2 (r_code.txt) – R function of AIC-based method. The R function for the AIC-based method is provided. An example analysis using the hypothetical vector is also provided.
